# Waist Circumference and Body Mass Index as Predictors of Disability Progression in Multiple Sclerosis: A Systematic Review and Meta-Analysis

**DOI:** 10.3390/jcm13061739

**Published:** 2024-03-18

**Authors:** Vasileios Giannopapas, Maria-Ioanna Stefanou, Vassiliki Smyrni, Dimitrios K. Kitsos, Maria Kosmidou, Sophia Stasi, Athanasios K. Chasiotis, Konstantina Stavrogianni, Georgia Papagiannopoulou, John S. Tzartos, George P. Paraskevas, Georgios Tsivgoulis, Sotirios Giannopoulos

**Affiliations:** 1 Second Department of Neurology, Attikon University Hospital, National and Kapodistrian University of Athens, 12462 Athens, Greece; bgiannopapas@gmail.com (V.G.); marianna421@hotmail.co.uk (M.-I.S.); b.smyrni@hotmail.com (V.S.); dkitsos@icloud.com (D.K.K.); thanosch1@gmail.com (A.K.C.); stavrogianni.k@gmail.com (K.S.); georgiapap22@hotmail.com (G.P.); jtzartos@gmail.com (J.S.T.); geoprskvs44@gmail.com (G.P.P.); tsivgoulisgiorg@yahoo.gr (G.T.); 2 Laboratory of Neuromuscular and Cardiovascular Study of Motion, University of West Attica, 12243 Aigaleo, Greece; 3 Department of Internal Medicine, Faculty of Medicine, University of Ioannina, 45110 Ioannina, Greece; mkosmid@uoi.gr; 4 Biomechanics Laboratory, Department of Physiotherapy, University of Peloponnese, 23100 Sparta, Greece; soniastasi1@gmail.com; 5 Department of Physiology, Faculty of Medicine, University of Ioannina, 45110 Ioannina, Greece

**Keywords:** multiple sclerosis, body mass index (BMI), waist circumference (WC), obesity, disability progression

## Abstract

**Background:** While obesity has been shown to elevate the risk of developing multiple sclerosis (MS), there is a lack of strong evidence regarding its role in the disability progression and status of MS patients. **Methods:** This systematic review and meta-analysis aimed to provide comparative estimates of WC and BMI in patients with MS (PwMS) and to investigate potential associations between the waist circumference (WC) and body mass index (BMI) and demographic and specific MS characteristics. Adhering to PRISMA guidelines, a detailed search of the MEDLINE PubMed, Cochrane Library, and Scopus databases was conducted. **Results:** A total of 16 studies were included. The pooled mean WC and BMI among PwMS was estimated to be 87.27 cm (95%CI [84.07; 90.47]) and 25.73 (95%CI [25.15; 26.31]), respectively. Meta-regression models established a significant bidirectional relationship between WC and the Expanded Disability Scale (EDSS) (*p* < 0.001) but not between BMI and EDSS (*p* = 0.45). Sensitivity analyses showed no association between WC and age (*p* = 0.48) and a tendency between WC and disease duration (*p* = 0.08). **Conclusions:** Although WC measurements classify PwMS as normal weight, BMI measurements classify them as overweight. Therefore, WC should complement BMI evaluations in clinical practice. Additionally, our findings highlight the significant association between abdominal fat, as indicated by WC, and disease progression. Considering the heightened risk of cardiovascular comorbidity and mortality among PwMS, we recommend integrating both WC and BMI as standard anthropometric measurements in routine clinical examinations and targeted prevention strategies for PwMS.

## 1. Introduction

Multiple sclerosis (MS) is the most common immune-mediated, inflammatory demyelinating condition of the central nervous system (CNS), characterized by progressive neurodegeneration, gliosis, and neuroinflammation [1]. Common symptomatology ranges from muscle weakness, hypertonia, sensory deficits, cognitive decline, and bladder and bowel problems to sexual dysfunction [1]. In the last decade, there has been an increased research interest in the role of comorbid cardiovascular conditions in the progression of disability in people with MS (PwMS). More specifically, common cardiovascular comorbidities, including hypertension, type 2 diabetes mellitus (T2DM), dyslipidemia, and obesity, have been shown to increase cumulative disability and disability progression [2,3,4]. In addition, obesity and a high body mass index (BMI) have been associated with increased odds for myocardial infraction, acute ischemic stroke, metabolic syndrome, and T2DM [5].

Furthermore, obesity has been shown to increase the incidence of MS in teenagers and young adults. In a recent study, the risk for developing MS in subjects whose BMI exceeded 27 kg/m^2^ at the age of 20 was two-fold higher than that in normal-weighted subjects of the same age [6]. Moreover, several mendelian randomization studies support the hypothesis that increased BMI and obesity influence a subject’s susceptibility to MS with the odds ranging from 1.21 to 1.4 [7,8,9]. Potential biological mechanisms still remain unknown, but two major hypotheses have been put forth: (a) chronic-low grade inflammatory response due to obesity; and (b) lower circulating levels of vitamin D in adults and children with high BMI [10,11]. Beyond the risk for developing MS, obesity has been shown to have a detrimental impact on MS progression. In PwMS with a comorbid obesity, decreased physical activity has been linked to the worsening of neuro-inflammation and cognitive decline and an increased risk of disability accrual [3,12,13].

To this day, there is a strong belief that PwMS have an increased risk of being overweight and/or obese, with preventive strategies being heralded as integral for the clinical management of MS. With respect to implemented anthropometric measures of obesity, cardiovascular research suggests that waist circumference (WC), compared to BMI, has a stronger association with metabolic risk in clinical practice [14]. Nevertheless, there is a dearth of evidence regarding the comparative efficacy of WC and BMI for the assessment of obesity and related disability in MS. Based on the previous considerations and given the clinical implication of comorbid obesity in PwMS, the aim of this systematic review and meta-analysis was two-fold. First, we sought to provide comparative estimates of the WC and BMI in PwMS, and second, we aimed to investigate potential associations between WC and BMI, including demographic and specific MS characteristics, including the Expanded Disability Status Scale (EDSS) and disease duration.

## 2. Methods

### 2.1. Standard Protocol, Approvals, and Registration

The pre-specified protocol of the present systematic review and meta-analysis is registered in the Open Search Framework (https://osf.io/xjtzf/, accessed on 19 December 2023). The methodology and results of the systematic review and meta-analysis are reported according to the Preferred Reporting Items for Systematic Reviews and Meta-Analyses (PRISMA), guidelines as well as the Meta-analysis of Observational Studies in Epidemiology proposal [15,16]. Due to the nature of the study design, no ethical board approval was required.

### 2.2. Data Sources, Search, and Study Selection

A systematic literature search was conducted to identify potentially eligible studies reporting on WC and BMI in PwMS by two independent reviewers (VG, MIS). The MEDLINE PubMed, Scopus, and Cochrane Library databases were searched using search strings that included the following search terms: “multiple sclerosis”, “BMI”, “overweight”, “waist circumference”. The complete search algorithm and PICO criteria are provided in the supplementary material. The search spanned from inception to 10 September 2023. Potential studies entailed randomized controlled trials or observational studies that included patients with a definite MS diagnosis, had a random sample selection, and reported WC values. We excluded (a) studies that included patients with clinically isolated syndrome, (b) studies that used BMI or WC as an inclusion criterion, (c) case reports, case series, commentaries, systematic reviews, non-peer reviewed studies, pre-prints, and conference abstracts, and (d) studies that reported outcomes that did not align with our inclusion criteria. All retrieved studies were independently assessed by two reviewers (VG, MIS), and any disagreements were resolved by the senior author (SG).

### 2.3. Quality Control, Bias Assessment, and Data Extraction

Eligible studies were assessed by two independent reviewers (VG, MIS) using the Risk of Bias for Non-Randomized Studies of Interventions (ROBINS-I) tool [17]. Potential disagreements were resolved via consensus. Data extraction from eligible studies included the following: (a) first author name, (b) study design, (c) year of publication, (d) total sample, (e) mean WC, (f) standard deviation (SD) for WC, (g) mean BMI, (h) SD for BMI, (i) mean age, (j) mean EDSS, (k) mean disease duration.

### 2.4. Outcomes

An aggregate data meta-analysis was performed with the inclusion of the identified studies.

The predefined primary outcome measures were twofold: (i) the association between the pooled mean WC and the pooled mean BMI and the patients’ disability status (EDSS) and (ii) the differences in WC and BMI between genders. Secondary outcomes included the following: (i) associations between age and WC and/or BMI; and (ii) the correlation between WC and disability progression in patients with MS.

### 2.5. Statistical Analysis

For the aggregate meta-analysis, the pooled mean value for the WC and BMI and 95% confidence intervals (95%CIs) were calculated using the random effect model of meta-analysis and the metamean function of the R-Meta package. Heterogeneity was assessed with the I^2^ and Cochran Q statistics [18,19]. For the qualitative interpretation of heterogeneity, I^2^ values > 50% and values > 75% were considered to represent substantial and considerable heterogeneity, respectively. The significance level for the Q statistic was set at 0.1. Publication bias across individual studies was graphically assessed when more than four studies were included in each analysis for the primary outcomes of interest, using funnel and radial plot inspection, as well as Egger’s linear regression test, and the equivalent z test for each pooled estimate with a two-tailed *p* value < 0.05 was considered statistically significant [20]. Potential associations between WC and/or BMI, including demographic or MS characteristics, were assessed based on meta-regression. In cases where the studies reported the median of a target outcome, the Quantile Estimation method was used to transform the median to a mean value [21]. All statistical analyses and figure production were carried out using RStudio for Windows [R studio/R Meta package v.7.0.0, GNU proect] [22].

### 2.6. Data Availability Statement

All data generated or analyzed during this study are included in this article and its Supplementary Information Files.

## 3. Results

### 3.1. Literature Search and Included Studies

**Figure 1:**
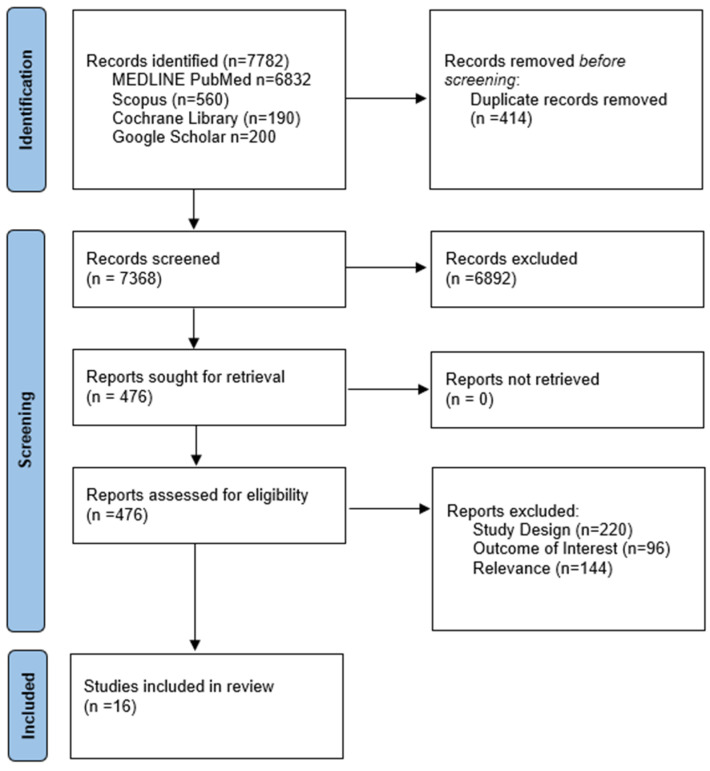
Prisma flowchart for the study selection.

The systematic database search yielded a total of 7782 records from the four databases. After excluding duplicates and out-of-scope records, 477 records were considered eligible and were assessed in full. After the full-text assessment, 461 records were excluded (study design, sample selection, reported outcomes) and 17 records with a total of 7962 PwMS were included (Figure 1).

### 3.2. Quality Control

The risk of bias was assessed using the ROBINS-I tool and is presented in Supplementary Figure S2, and it presented a moderate level of bias. Most studies presented significant biases due to confounding (i.e., by not reporting key confounding variables, such as MS subtypes, disability status, and disease duration).

### 3.3. Quantitative Analysis

#### 3.3.1. Primary Outcomes

**Figure 2:**
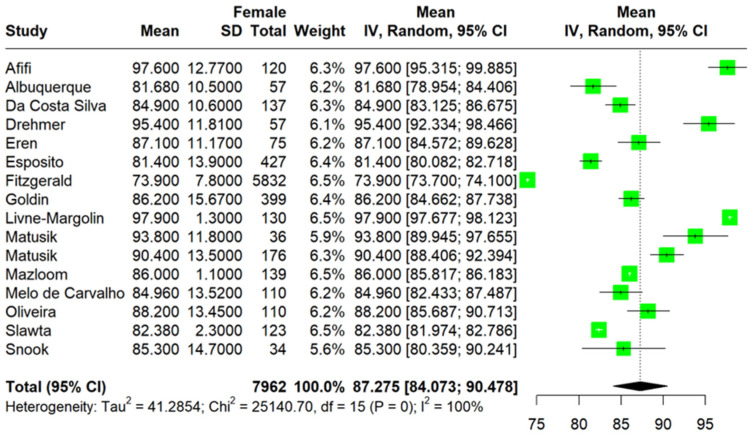
Forest plot: pooled mean WC values [23,24,25,26,27,28,29,30,31,32,33,34,35,36,37,38].

**Figure 3:**
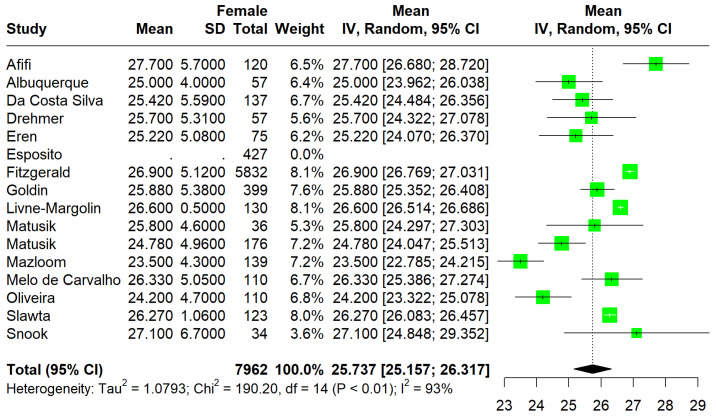
Forest plot: pooled mean BMI values [23,24,25,26,27,28,29,30,31,32,33,34,35,36,37,38].

A total of 16 [23,24,25,26,27,28,29,30,31,32,33,34,35,36,37,38] studies comprising a total of 7962 PwMS with a mean age of 42.1 years were included in the meta-analysis. Primary and secondary outcomes are summarized in Table 1. The sample consisted mainly of women with MS (77.4%) (Table 1). The pooled mean WC was 87.27 cm (95%CI: [84.07; 90.47], I^2^ = 100%, p_Q_ = 0) (Figure 2), while the mean BMI was 25.73 (95%CI [25.15; 26.31], I^2^ = 93%, p_Q_ < 0.01) (Figure 3). In a subset of 11 studies that reported the EDSS score, the pooled mean EDSS was 3.2 (95%CI: [2.54; 3.91], I^2^ = 99.8%, p_Q_ = 0) (min: 1.5, max: 5.5).

Utilizing a random effects meta-regression model, the bidirectional associations between EDSS and WC and BMI were assessed. A statistically significant association was detected between WC and EDSS (β = 4.01, SE = 1.09, *p* < 0.001) which translates to in “an increase of EDSS by 1 point resulting in a 4.01 cm increase of WC”. In the opposite direction, a statistically significant association was also noted between EDSS and WC (β = 0.15, SE = 0.04, *p* < 0.001), which translates to in “an increase of WC by 1 cm results in a 0.15-point increase of EDSS”. Regarding the relationship between BMI and EDSS, no statistically significant associations were uncovered in either direction (β = 0.14, *p* = 0.61 and β = 0.19, *p* = 0.45).

**Figure 4:**
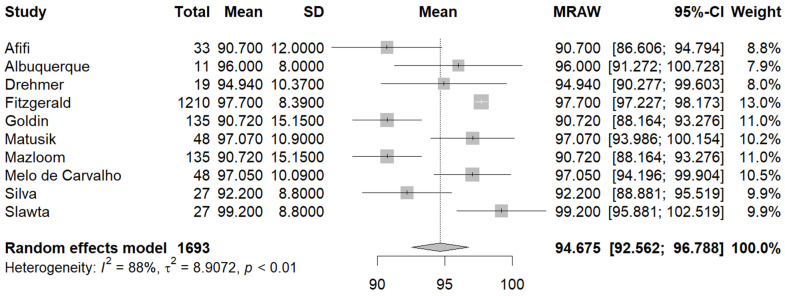
Forest plot: pooled mean WC values in the male subset [23,26,27,29,30,34,35,36,37,38].

**Figure 5:**
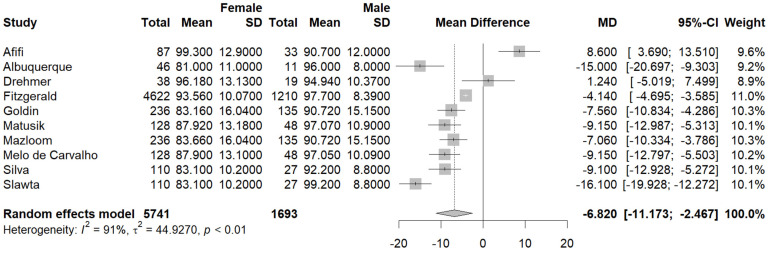
Forest plot: pooled mean WC values in the female subset [23,26,27,29,30,34,35,36,37,38].

**Figure 6:**
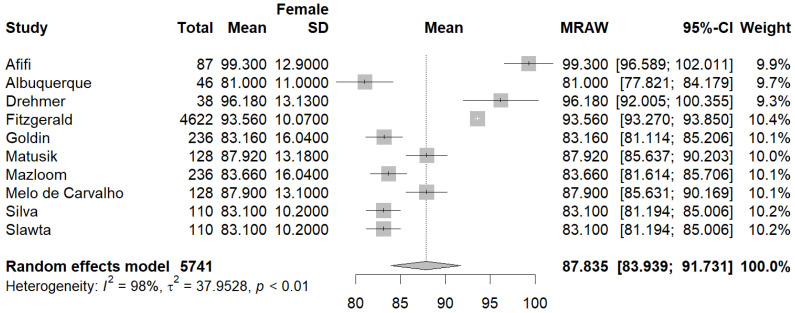
Forest plot: mean difference in WC between male and female PwMS [23,26,27,29,30,34,35,36,37,38].

Ten studies [23,26,27,29,30,34,35,36,37,38] reported the mean WC in the male and female population. The pooled mean WC in the male subgroup was 94.67 cm (95% CI [92.56, 96.78], I^2^ = 88%, p_q_ = < 0.01) (Figure 4), and the pooled mean WC in the female subgroup was 87.83 cm (95% CI [83.93, 91.73], (I^2^ = 98%, p_q_ < 0.01) (Figure 5). A statistically significant difference was also found between the mean WC between female and male PwMS, with female PwMS having a mean difference of −6.81 cm (95%CI [−11.17, −2.46], I^2^ = 91.3%, p_z_ = 0.00) (Figure 6) compared to male PwMS.

**Figure 7:**
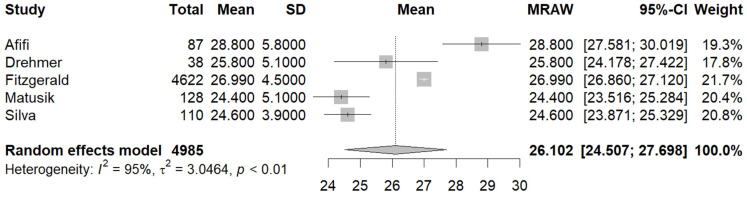
Forest plot: pooled mean BMI values in the male subset [27,29,30,35,37].

**Figure 8:**
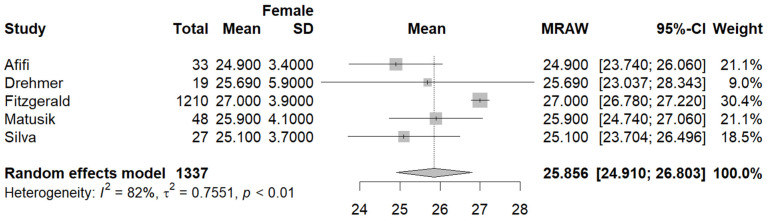
Forest plot: pooled mean BMI values in the female subset [27,29,30,35,37].

Five studies [27,29,30,35,37] reported the mean BMI in the male and female population. The pooled mean BMI in the male subgroup was 26.10 (95%CI [24.50, 27.69], I^2^ = 95%, *p* < 0.01) (Figure 7), while in the female population, the pooled mean BMI was 25.85 (95%CI [24.91, 26.82], I^2^ = 82%, *p* < 0.01) (Figure 8).

#### 3.3.2. Secondary Outcomes

Subsequent sensitivity analyses were performed to identify potential associations among WC, BMI, and demographic and MS characteristics after the exclusion of studies that did not report the corresponding measurements. Patient age did not have a statistically significant association with WC (*p* = 0.48). In the case of BMI, there was a statistically significant association between BMI and age (β = 0.06, *p* = 0.04), which translates to “an increase in age by 1 year leads to a 0.06 increase in BMI”. Furthermore, there was strong trend towards an association between the mean WC and disease duration, which did not reach statistical significance (β = 0.84, se = 0.49, *p* = 0.08). Contrarily, BMI had no association with disease duration (β = −0.05, *p* = 0.63)

Finally, five studies reported a correlation index between EDSS and WC, with the pooled correlation being 0.17 (95%CI [0.07–0.27], *p* < 0.001), which is indicative of a weak positive correlation between WC and EDSS.

#### 3.3.3. Publication Bias

Publication bias was assessed through funnel plot inspection for the primary outcomes. Both funnel plots (Supplementary Figures S6 and S7) appeared to be asymmetrical and indicative of high publication bias (corresponding results from Egger’s linear testing for publication β = 2.40, t = 0.21, *p* = 0.83 and β = −2.37, t = −2.55, *p* = 0.02 respectively).

## 4. Discussion

In the present systematic review and meta-analysis, the pooled mean WC among PwMS was estimated at 87.27 cm (95%CI [84.07; 90.47]), with the corresponding pooled mean BMI being 25.73 (95%CI [25.15; 26.31]). When stratifying by gender, female PwMS had a mean WC of 87.83 cm (95% CI [83.93, 91.73]), while male PwMS had a statistically significantly higher mean WC by 6.81 cm (corresponding to 94.67 cm (95% CI [92.56, 96.78]). Based on the ranges of the WC and BMI in the normal population [39,40,41], our findings indicate that PwMS fall within the normal range when evaluated based on the WC but in the overweight range when evaluated based on the BMI. This discrepancy may be due the fact that BMI is a function of weight and height, whereas WC does not account for body stature and may thus be complementary to BMI for obesity assessments in clinical practice. To overcome the inherent limitations of both BMI and WC, the waist-to-height ratio has been recently introduced as an index for central adiposity with improved performance in predicting cardiometabolic risk. Nevertheless, waist-to-height data in the PwMS population were not available for meta-analysis.

The clinical implications of WC and BMI assessments were further explored, analyzing the association between disability and the two different anthropometric evaluation tools. EDSS, as the main clinical disability index for PwMS, was found to have a weak positive correlation with WC. Additionally, our results indicated a bidirectional relationship between EDSS and WC. More specifically, a 1-point increase in the EDSS score was shown to result in a 4 cm increase. Accordingly, a 1 cm increase in the WC resulted in a 0.15-point increase in the EDSS. In our view, lower physical activity and increased levels of disability in PwMS, which are directly associated with an increase in visceral fat deposits, explain, in part, the bidirectional relationship between the EDSS and WC. Lastly, although there was a significant association between the level of disability (EDSS score) and WC, no association was found between BMI and disability progression in PwMS.

The positive bi-directional association between WC and disability progression in PwMS could be attributed to physical deconditioning resulting from reduced mobility, fatigue, and physical inactivity. More specifically, when compared to healthy individuals, the wide majority of PwMS have been shown to have reduced aerobic capacity [42], normal-to-low maximal exercise capacity [43], and significantly decreased walking endurance. Additional studies highlight that a lack of physical exercise combined with physical deconditioning acts as a contributing factor to the reduced walking capacity observed in PwMS. On this note, the role of chronic fatigue should not be overlooked, as it is an equally important contributing factor related to the physical inactivity and reduced mobility seen in these patients [44]. More specifically, chronic fatigue, which is a frequently occurring phenomenon in PwMS with mild disability, has been linked to physical deconditioning, which, in turn, has been shown to be associated with increased neuromuscular fatigability, and, in turn, reduced walking capacity [45]. Accordingly, active participation in physical exercise activities in PwMS has been shown to have a gradual or non-linear heart rate response that is analogous to that in healthy individuals, suggesting that physiological deconditioning is linked to MS-related autonomic dysfunction [43].

Several considerations should be taken into account when interpreting the previous findings. To begin, research has shown that the influence of genetic and environmental factors associated with a high BMI contribute to MS development [6,7]. More specifically, a BMI > 27 kg/m^2^ during puberty and early adolescence is associated with a significantly increased risk of MS development [6,7]. Moreover, research has proposed the existence of a possible underlying mechanism between obesity and an increased risk of developing MS, mediated by the actions of the immune system and hormonal factors. More specifically, studies propose that obesity, as expressed by an increased BMI, directly influences the immune system via the actions of specific hormones, including adiponectin and leptin, thereby fostering a pro-inflammatory environment [6,7]. Despite the association between a higher BMI and increased susceptibility to MS development, however, there are conflicting data in the literature concerning the correlation between BMI and disease progression and disability in PwMS.

Furthermore, when compared to BMI, WC has a distinctive advantage as a prognostic index, due to its sensitivity in detecting abdominal obesity, which has been shown to be predictive of cardiovascular pathology risk and type 2 Diabetes Mellitus (T2DM) even in individuals with a normal BMI [45]. Notably, research indicates that WC more accurately predicts cardiovascular risk and T2DM in PwMS compared to the BMI, which underestimates adiposity in these patients [46,47]. Moreover, multiple studies have demonstrated a significant association between an increased WC and elevated risks of cerebral small vessel disease, T2DM, and neurological disability [48,49]. Consequently, WC emerges as a more accurate prognostic indicator of the cardiovascular burden in PwMS, which has been linked to an elevated risk of cerebral small vessel disease and neurological disability.

Given the increased risk of cardiovascular comorbidities in PwMS and their detrimental impact on the overall disease severity and disability progression, WC and BMI in the upper normal ranges can still have a negative effect on disease progression and the overall quality of life of PwMS and are correlated with the patient’s disability levels [27,28,32,36]. Concerning the potential underlying pathophysiological mechanisms, several pathways appear to be linking obesity and neuroinflammation.

On the one hand, fat tissue has been found to be associated with the overproduction of pro-inflammatory adipokines and, on the other hand, with the attenuation of anti-inflammatory adipokines [50]. Adipokines, which are soluble factors secreted by adipose tissue, play diverse roles in biological processes and are implicated in the chronic inflammatory state associated with obesity [50]. A recent study by Breden and colleagues suggests a clear gender dichotomy, with obese female individuals having higher circulating levels of total and high-molecular-weight adiponectin and leptin serum concentrations [51]. They impact immune function, metabolism, and the nutritional status. The lean adipose tissue stroma is comprised of regulatory T cells (Treg cells), invariant natural killer cells (iNKT cells), M2 macrophages, natural killer cells (NK cells), innate lymphoid cells type 2 (ILC2), and eosinophils, collectively fostering an anti-inflammatory milieu [50]. Obesity alters the balance of the anti-inflammatory environment, promoting the creation of a pro-inflammatory state characterized by a substantial increase in M1 macrophages and neutrophils, CD8+ T cells, and T helper 1 cells [50]. Simultaneously, there is a decrease in Treg cells, ILC2 cells, iNKT cells, and Th2 immunosuppressive mediators (such as IL-4, IL-10, and TGF-β), combined with the decreased activation of peroxisome proliferator-activated gamma (PPAR-γ), crucial for sustaining the homeostasis of the adipose tissue [50]. Collectively, these factors result in a chronic low-grade inflammatory state, disrupting both local and systemic immune system regulation, increasing the susceptibility to and progression of autoimmune disorders, such as MS, while exhibiting fluctuations depending on patient age, age at MS onset, and early vs. late stages of MS [50].

### 4.1. Issues to Be Addressed

Following the discussion above, several factors have been identified to be addressed in future research. First, there is a need for a more consistent measurement of WC and height. Over the course of MS, especially in patients with higher disability levels, there is an increased chance of musculoskeletal disorders that may affect height measurements and/or protrusion (in PwMS that use a wheelchair), which may result in measurement discrepancies.

Second, while BMI remains a primary measure of obesity in clinical settings, emerging evidence underscores its inadequacy as a reliable estimator of obesity. Findings from studies involving patients with spinal cord injuries support our hypothesis that BMI has a reduced efficacy in explaining the variance in measured percent fat mass compared to individuals without such injuries [52,53]. These limitations can be attributed to potential measurement error and BMI’s inherent inability to differentiate between fat and fat-free mass, as well as its inability to accurately assess body fat distribution. In our opinion, a combination of anthropometric measurements, including height, weight, waist circumference, the waist-to-height ratio, and bioelectrical impedance analysis should be recorded for each patient with MS.

Taking into consideration the information discussed above and the recent finding of Lutfullin and colleagues that suggest that the presence of obesity at MS onset is correlated with higher disease severity and poorer outcomes [54], we suggest that WC should be used as a complementary assessment measure in clinical settings for various reasons. First, WC is a measure of abdominal and visceral obesity, thereby accurately differentiating between the fat distribution patterns seen in PwMS, as opposed to BMI, which measures the overall body fat solely based on an individual’s weight and height. Second, since visceral fat deposition is closely related to cardiovascular factors, which may worsen MS symptom severity and disease progression, using WC in clinical practice could be a more representative, and possibly reliable, measure of visceral fat changes in these patients. Third, bearing in mind the potential of WC for detecting fat distribution alterations, which result from decreased kinesis and changes in patients’ metabolism, it may be a more representative monitoring tool of body fat distribution in the long-term. Lastly, this study established a positive relationship between WC and EDSS, thereby further emphasizing its potential use as an additional monitoring tool for disease severity.

### 4.2. Limitations

To our knowledge this is the first study that attempts to estimate the mean WC of PwMS. Nonetheless, our study is not without limitations. First, there is a high degree of heterogeneity among included studies in terms of methodology, population characteristics, and reported outcomes. Second, the analyzed sample is small and relatively young with a low degree of disability (in studies reporting EDSS). Third, concomitant disease-modifying therapies, comorbidities, and further confounders could not be included in the meta-regression (as only aggregate data were reported in the included studies). Fourth, as subgroup analyses based on the MS type could not be performed due to data unavailability, our results warrant replication for different MS patient populations in future studies.

## 5. Conclusions

The results of this study suggest that PwMS are in the normal-weight category based on WC but in the overweight category based on BMI. Therefore, WC should be used complementary to BMI assessments in clinical practice. In addition, our findings suggest that abdominal fat, as reflected by WC, is significantly associated with disability progression. Taking the increased risk of cardiovascular comorbidity and mortality of PwMS into account, we suggest that WC and BMI, as standard anthropometric measurements, are both implemented in routine clinical examination and in targeted prevention strategies for PwMS.

## Figures and Tables

**Table 1:** Included studies.

Author	Year	Region	Study Design	Sample Size	Mean Age	RRMS	EDSS	Disease Duration	Female/Male
Slawta et al. [23]	2002	USA	OBS	123	46.4				123/0
Snook et al. [24]	2005	USA	OBS	34	44.3			6.9	32/2
Oliveira et al. [25]	2014	Brazil	RCT	110	38.2	64.7	3.2	6.3	75/35
Mazloom et al. [26]	2017	Italy	CS	139	26.5				118/21
(Da Costa) Silva et al. [27]	2018	Brazil	CS	137		88.3%			110/27
Matusik et al. [28]	2019	Poland	OBS	36			4.4		24/12
Drehmer et al. [29]	2020	Spain	OBS	57	49.7		3.8		38/19
Fitzgerald et al. [30]	2020	USA	OBS	5832	54.6				4622/1210
Esposito et al. [31]	2020	Italy	CS	427	42.4		3.6	10	292/135
Livne-Margolin et al. [32]	2021	Israel	CS	130	55.8		5.5	18.2	94/36
Eren et al. [33]	2021	Turkey	RCT	75	38.4	70.7%	2.5	7.82	50/25
Albuquerque et al. [34]	2021	Brazil	CC	57	34.6	89.5%	1	6	48/9
Matusik et al. [35]	2022	Poland	OBS	176	45.7	69.8%	3.3	10.9	128/48
Goldin et al. [36]	2023	German	Cohort	399	41.8				236/135
Afifi et al. [37]	2023	Egypt	RCT	120	33.5		3.1	7	87/33
Melo de Carvalho et al. [38]	2023	Brazil	OBS	110	37.1	89.1%	1.9	6.29	89/21

Note. EDSS and disease duration are presented as mean values. OBS: observational study, RCT: randomized control trial, age is reported in years. RRMS: relapsing remitting multiple sclerosis CC: case-control study.

## Data Availability

All data are presented in the main text and Supplementary Materials.

## Supplementary Materials

The following supporting information can be downloaded at: https://www.mdpi.com/article/10.3390/jcm13061739/s1, Figure S1: Traffic light plot of ROBINS-I tool; Figure S2: Summary of bias analysis using the ROBINS-I tool; Figure S3: Forest plot: pooled mean WC in the male subgroup; Figure S4: Forest plot: pooled mean WC in the female subgroup; Figure S5: Forest plot: mean difference in WC between male and female PwMS; Figure S6: Funnel plot: pooled mean WC; Figure S7: Funnel plot: pooled mean BMI.
