# Supplementary material for: Waist Circumference and Body Mass Index as Predictors of Disability Progression in Multiple Sclerosis: A Systematic Review and Meta-Analysis
Source: J Clin Med. 2024 Mar 18;13(6):1739. doi: 10.3390/jcm13061739 (PMC10970884; doi:10.3390/jcm13061739)

Supplementary materials

No of Tables :0

No of Figures: 7

Figures Legend

Figure S1 : Traffic light plot ROBINS-I tool

Figure S2: Summary of Bias Analysis using the ROBINS-I tool

Figure S3: Forest plot : Pooled WC mean in the male subgroup

Figure S4: Forest plot : Pooled WC mean in the female subgroup

Figure S5: Forest plot : Mean difference of WC in between male and female PwMS

Figure S6: Funnel plot : Pooled WC mean

Figure S7: Funnel plot : Pooled BMI mean

Pico Criteria

P: Adults with a definite diagnosis of Multiple Sclerosis

I: n/a

C:n/a

O: BMI, Waist circumference

Search algorithm

Pubmed: (("multiple sclerosis"[Title/Abstract] AND "waist circumference"[Title/Abstract]) AND"bmi"[Title/Abstract] OR "overweight"[Title/Abstract]) AND ((medline[Filter]) AND (fha[Filter]) AND (observationalstudy[Filter] OR randomizedcontrolledtrial[Filter]) AND (humans[Filter]) AND (alladult[Filter]))

Scopus: (multiple AND sclerosis) AND ((waist AND circumference) OR bmi OR overweight)

Cochrane Library:(multiple AND sclerosis) AND ((waist AND circumference) OR bmi OR overweight)

Figure S1

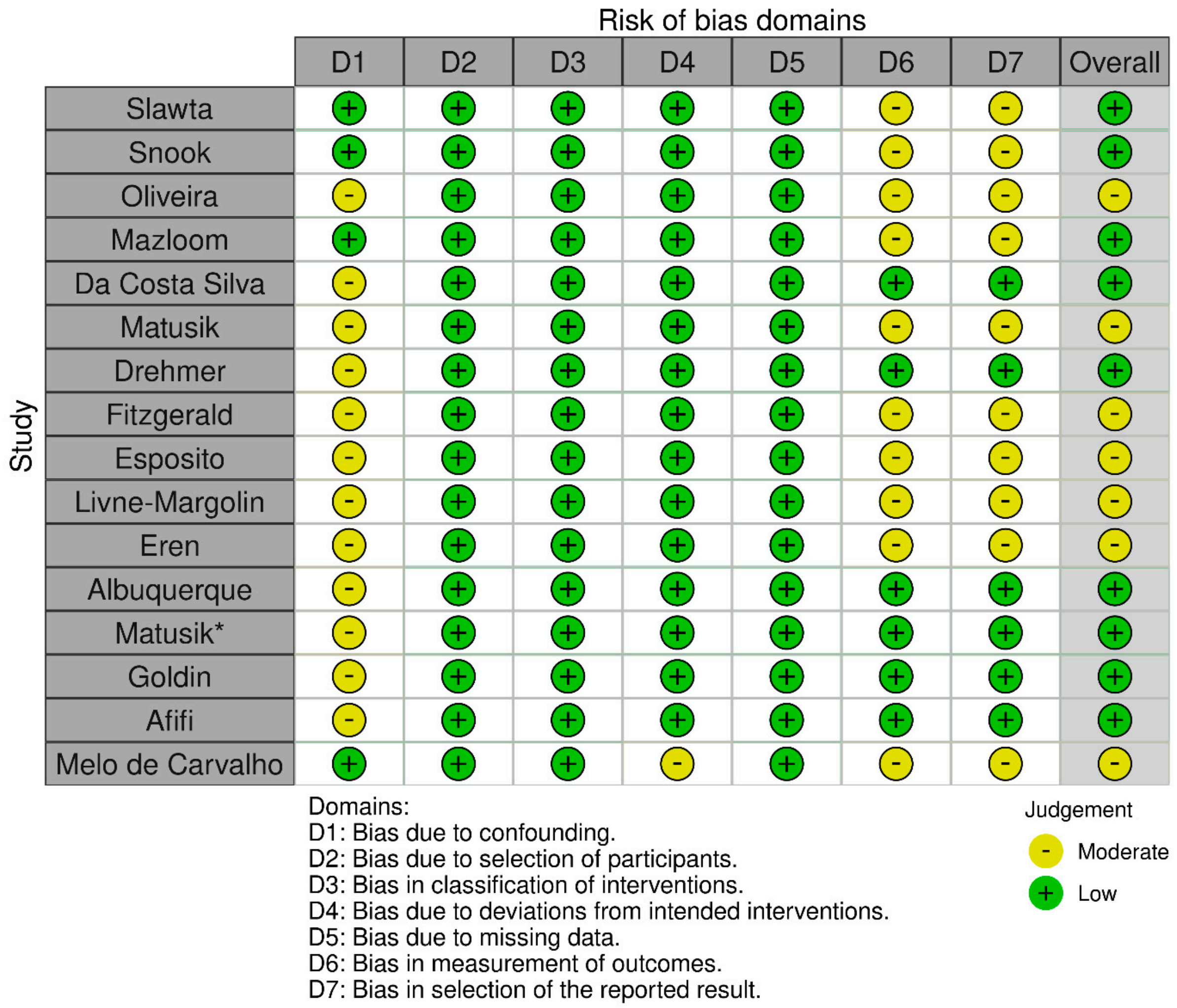

Figure S2

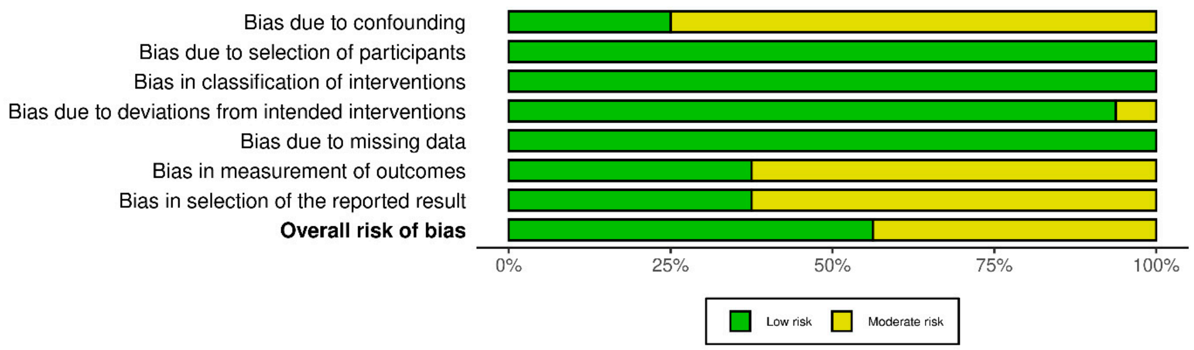

Figure S3

Study	Mean	SD	Total	Weight (common)	Weight (random)	Mean IV, Fixed + Random, 95% CI
Da Silva	99.200	8.8000	27	17.8%	17.9%	99.200 [95.881; 102.519]
Drehmer	94.940	10.3700	19	9.0%	14.1%	94.940 [90.277; 99.603]
Albuquerque	96.000	8.0000	9	7.2%	12.7%	96.000 [90.773; 101.227]
Matusik	97.050	10.0900	48	24.1%	19.3%	97.050 [94.196; 99.904]
Goldin	90.720	15.1500	135	30.0%	20.2%	90.720 [88.164; 93.276]
Afifi	93.500	11.9000	33	11.9%	15.8%	93.500 [89.440; 97.560]
**Total (common effect, 95% CI)**			**271**	**100.0%**	**--**	**94.844 [93.443; 96.244]**
**Total (random effect, 95% CI)**			**--**		**100.0%**	**95.167 [92.494; 97.839]**

Heterogeneity: $Tau^2 = 7.5107$; $Chi^2 = 19.52$, df = 5 ($P < 0.01$); $I^2 = 74\%$

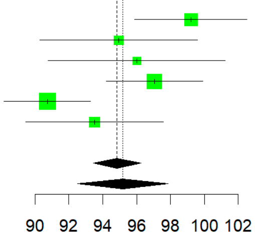

Figure S4

Study	Mean	SD	Total	Weight (common)	Weight (random)	Mean IV, Fixed + Random, 95% CI
Da Silva	83.100	10.2000	110	27.5%	17.0%	83.100 [81.194; 85.006]
Drehmer	96.180	13.1300	38	5.7%	16.0%	96.180 [92.005; 100.355]
Albuquerque	81.000	11.0000	48	10.3%	16.5%	81.000 [77.888; 84.112]
Matusik	87.900	13.1000	128	19.4%	16.9%	87.900 [85.631; 90.169]
Goldin	83.660	16.0400	236	23.9%	17.0%	83.660 [81.614; 85.706]
Afifi	99.200	13.1000	87	13.2%	16.7%	99.200 [96.447; 101.953]
**Total (common effect, 95% CI)**			**647**	**100.0%**	**--**	**86.821 [85.822; 87.821]**
**Total (random effect, 95% CI)**			**--**		**100.0%**	**88.431 [82.454; 94.409]**

Heterogeneity: $Tau^2 = 53.7588$; $Chi^2 = 135.11$, df = 5 ($P < 0.01$); $I^2 = 96\%$

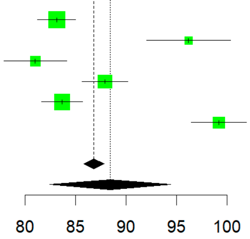

Figure S5

Study	Experimental Mean	SD	Total	Control Mean	SD	Total	Weight	Mean Difference IV, Random, 95% CI
Da Silva	83.100	10.2000	110	99.200	8.8000	27	17.2%	-16.100 [-19.928; -12.272]
Drehmer	96.180	13.1300	38	94.940	10.3700	19	15.8%	1.240 [-5.019; 7.499]
Albuquerque	81.000	11.0000	48	96.000	8.0000	9	15.9%	-15.000 [-21.083; -8.917]
Matusik	87.900	13.1000	128	97.050	10.0900	48	17.2%	-9.150 [-12.797; -5.503]
Goldin	83.660	16.0400	236	90.720	15.1500	135	17.4%	-7.060 [-10.334; -3.786]
Afifi	99.200	13.1000	87	93.500	11.9000	33	16.6%	5.700 [0.795; 10.605]
**Total (95% CI)**			**647**			**271**	**100.0%**	**-6.806 [-13.701; 0.088]**

Heterogeneity: $Tau^2 = 68.3301$; $Chi^2 = 61.28$, df = 5 ($P < 0.01$); $I^2 = 92\%$

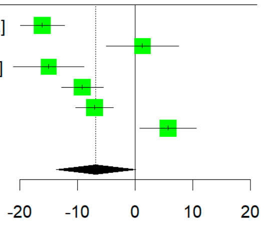

Figure S6

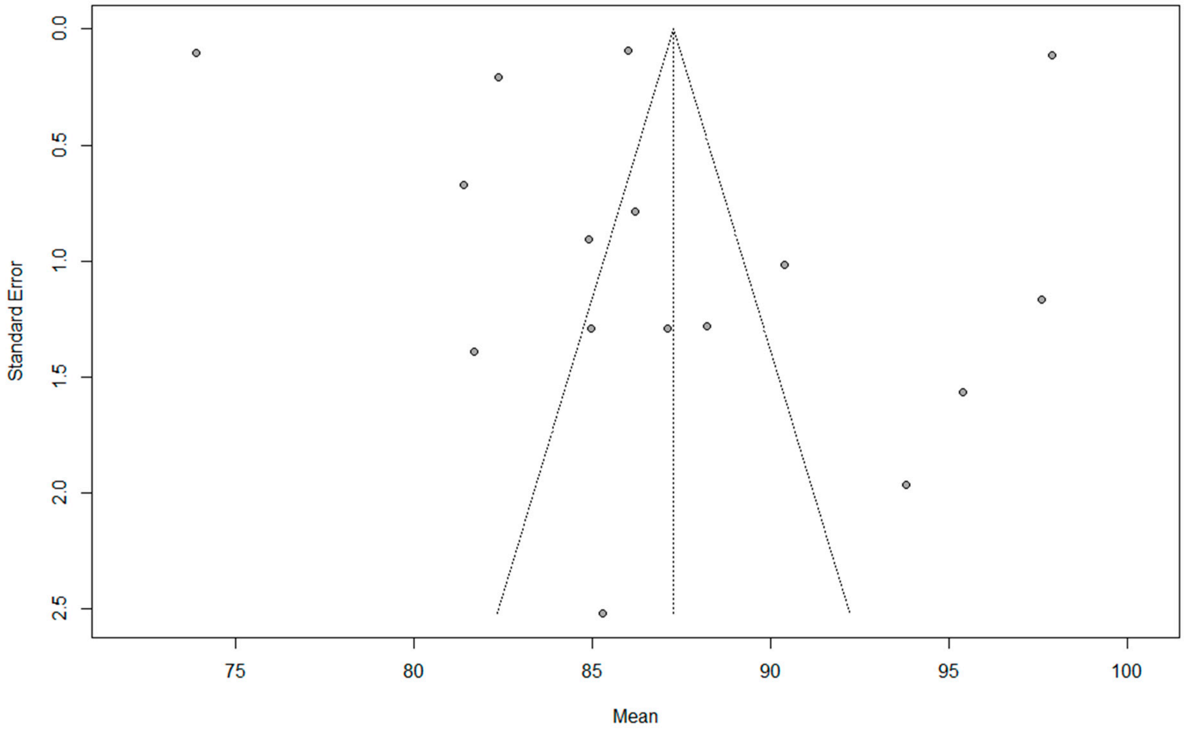

Figure S7

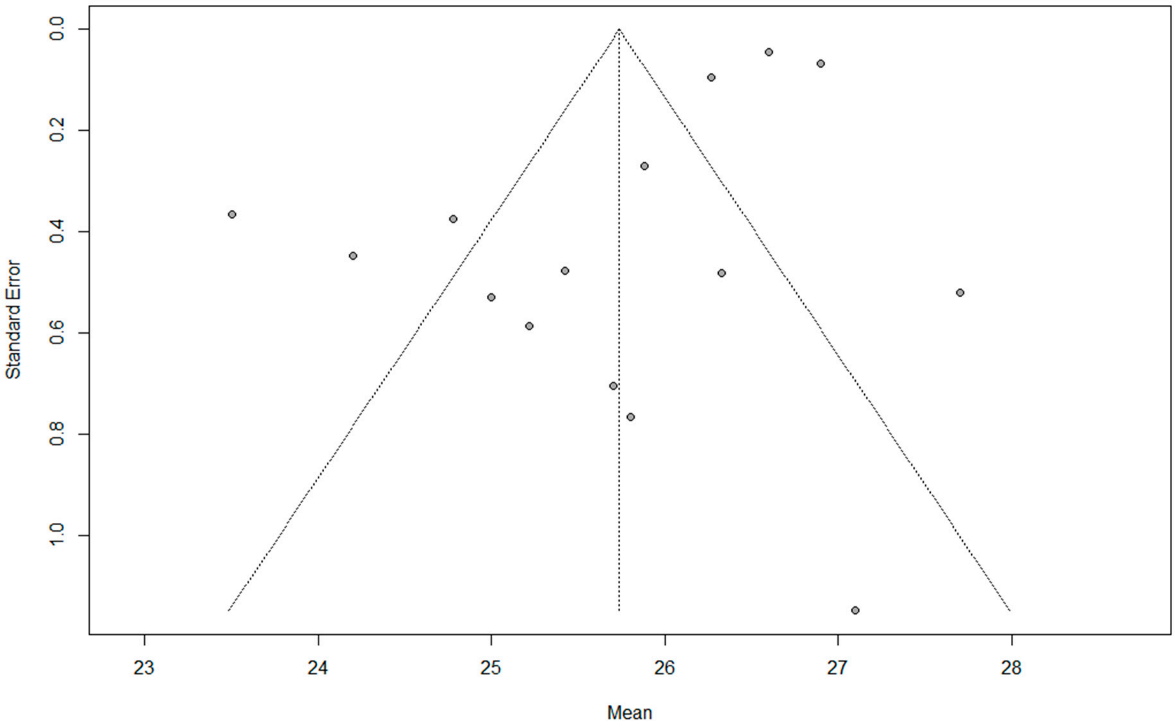

Supplement: Supplementary file 1 [file jcm-13-01739-s001.zip › jcm-2897560-supplementary.pdf]
